# Natural climate solutions for Canada

**DOI:** 10.1126/sciadv.abd6034

**Published:** 2021-06-04

**Authors:** C. Ronnie Drever, Susan C. Cook-Patton, Fardausi Akhter, Pascal H. Badiou, Gail L. Chmura, Scott J. Davidson, Raymond L. Desjardins, Andrew Dyk, Joseph E. Fargione, Max Fellows, Ben Filewod, Margot Hessing-Lewis, Susantha Jayasundara, William S. Keeton, Timm Kroeger, Tyler J. Lark, Edward Le, Sara M. Leavitt, Marie-Eve LeClerc, Tony C. Lemprière, Juha Metsaranta, Brian McConkey, Eric Neilson, Guillaume Peterson St-Laurent, Danijela Puric-Mladenovic, Sebastien Rodrigue, Raju Y. Soolanayakanahally, Seth A. Spawn, Maria Strack, Carolyn Smyth, Naresh Thevathasan, Mihai Voicu, Christopher A. Williams, Peter B. Woodbury, Devon E. Worth, Zhen Xu, Samantha Yeo, Werner A. Kurz

**Affiliations:** 1 Nature United, Ottawa, ON, Canada.; 2 The Nature Conservancy, Arlington, VA, USA.; 3 Smithsonian Conservation Biology Institute, Front Royal, VA, USA.; 4 Agriculture and Agri-Food Canada, Indian Head, SK, Canada.; 5 Ducks Unlimited Canada, Institute for Wetland and Waterfowl Research, Stonewall, MB, Canada.; 6 McGill University, Montreal, QC, Canada.; 7 University of Waterloo, Waterloo, ON, Canada.; 8 Agriculture and Agri-Food Canada, Ottawa, ON, Canada.; 9 Canadian Forest Service, Natural Resources Canada, Victoria, BC, Canada.; 10 The Nature Conservancy, Minneapolis, MN, USA.; 11 University of Toronto, Toronto, ON, Canada.; 12 Hakai Institute, Quadra Island, BC, Canada.; 13 University of Guelph, Guelph, ON, Canada.; 14 University of Vermont, Burlington, VT, USA.; 15 University of Wisconsin-Madison, Madison, WI, USA.; 16 Canadian Forest Service, Natural Resources Canada, Ottawa, ON, Canada.; 17 Canadian Forest Service, Natural Resources Canada, Toronto, ON, Canada.; 18 Canadian Forest Service, Natural Resources Canada, Edmonton, AB, Canada.; 19 Viresco Solutions, Victoria, BC, Canada.; 20 University of British Columbia, Vancouver, BC, Canada.; 21 Clark University, Worcester, MA, USA.; 22 Cornell University, Ithaca, NY, USA.

## Abstract

Alongside the steep reductions needed in fossil fuel emissions, natural climate solutions (NCS) represent readily deployable options that can contribute to Canada’s goals for emission reductions. We estimate the mitigation potential of 24 NCS related to the protection, management, and restoration of natural systems that can also deliver numerous co-benefits, such as enhanced soil productivity, clean air and water, and biodiversity conservation. NCS can provide up to 78.2 (41.0 to 115.1) Tg CO_2_e/year (95% CI) of mitigation annually in 2030 and 394.4 (173.2 to 612.4) Tg CO_2_e cumulatively between 2021 and 2030, with 34% available at ≤CAD 50/Mg CO_2_e. Avoided conversion of grassland, avoided peatland disturbance, cover crops, and improved forest management offer the largest mitigation opportunities. The mitigation identified here represents an important potential contribution to the Paris Agreement, such that NCS combined with existing mitigation plans could help Canada to meet or exceed its climate goals.

## Teaser

Canada has natural solutions to support its efforts to tackle climate change: forests, wetlands, grasslands, and agriculture.

## INTRODUCTION

Canada’s landscapes are already experiencing the effects of climate change, including the thawing of permafrost that releases greenhouse gases (GHGs), shifts in ecotones and species composition, and increases in tree mortality from drought, wildfire, and insect outbreaks (*1*, *2*). In central Canada, rising temperatures are linked to an increase in multiple-day precipitation events (*3*) and recent large floods that have affected key agricultural areas (*4*). To reduce further damage in Canada and beyond, we need to reduce emissions from fossil fuels while increasing removals and decreasing emissions of GHG associated with land sector activities (*5*, *6*).

Natural climate solutions (NCS) are a suite of protection, improved management, and restoration actions (“pathways”) in forests, grasslands, agricultural areas, and wetlands that provide additional climate mitigation beyond business as usual (BAU). Unlike other nascent carbon capture technologies, NCS are broadly scalable and deployable now (*7*) and provide many co-benefits beyond climate mitigation (table S1). Previous studies estimate that full implementation of all cost-effective NCS, including avoided conversion and restoration of natural lands and improved management of working lands, can provide up to one-third of the global mitigation needed in 2030 to keep warming below 2°C (*7*) and, in the United States, could mitigate up to 21% of net annual emissions (*8*). Underlying these estimates is substantial variation in the mitigation potential that depends on both geography and the type of action. Global efforts must substantially increase to prevent catastrophic warming (*9*), and urgent action requires refined estimates of the potential for NCS that incorporate nationally specific biophysical constraints and feasibility considerations. Here, we quantify how individual NCS can help realize and augment targets for emission reductions in Canada, which is especially critical given that the activities currently in the Pan-Canadian Framework on Clean Growth and Climate Change will not be sufficient to achieve Canada’s goals under the Paris Agreement (*10*, *11*).

We consider 24 distinct pathways of NCS that are carefully defined to avoid double counting (Table 1). While our principal focus is to estimate the mitigation potential of NCS at a national scale, we provide subnational estimates where possible (Table 2). For each pathway, we quantify the potential for annual mitigation in 2030 and cumulative mitigation between 2021 and 2030. By 2030, Canada’s Nationally Determined Contribution (NDC) to the Paris Agreement aims to reduce economy-wide GHG emissions to 511 Tg CO_2_ equivalents (CO_2_e)/year (i.e., 30% below 2005 levels) (*5*). Furthermore, the Canadian government has committed to achieve net zero emissions by 2050 (*5*). We thus track the mitigation potential of all NCS to 2050, as NCS maintain or increase mitigation beyond our 2030 implementation window.

**Table 1:** Pathway definitions for NCS.

**Natural climate solution**	**Definition**
Cover crops	Increased sequestration of CO_2_e into agricultural soils from growing additional cover crops in late summer-fall with or after the cash crop, in early spring before planting the cash crop, or on fallow areas. We model additional adoption of cover crops over 20.5 Mha (63% of land in Canada with annual cash crops) where growing conditions and cash crop type did not preclude cover crop adoption. Mitigation includes soil organic carbon storage, as well as avoided direct and indirect N_2_O emissions from the field and avoided emissions from fertilizer manufacture where relevant (see the Supplementary Materials).
Crop residue – biochar	Increased sequestration of CO_2_ in soil carbon by amending agricultural soils with biochar produced by converting crop residue to recalcitrant carbon (i.e., charcoal) through pyrolysis. We limit the source of biochar production to crop residue that can be sustainably harvested. We assume that biochar carbon persists longer than 100 years and has no effects on soil emissions of N_2_O or CH_4_. We also evaluate the alternative use of crop residue to produce bioethanol but highlight the biochar option in the main text (see the Supplementary Materials for rationale).
Nutrient management	Avoided N_2_O emissions (reported in CO_2_e) due to implementation of the “4R” best practices (right source, right rate, right time, and right place) for use of nitrogen fertilizer. We project BAU growth in fertilizer use in Canada as 2.8% annually by 2030 and then assess additional mitigation of a full 4R implementation scenario compared to this BAU. We account for avoided N_2_O emissions in the field due to more efficient use of nitrogen fertilizer and avoided emissions from fertilizer manufacture.
Tree intercropping (trees in agricultural lands pathway)	Increased CO_2_e sequestration from additional trees planted in rows among crop and hay lands. We include carbon storage in above- and belowground biomass and soil, as well as deductions for the albedo effect of transitioning from crop or hay to partial deciduous cover of 111 trees/ha. We model the expansion of tree intercropping across all crop and hay lands with class 3 soils in Ontario and Quebec (797,298 ha), because these soils have moderate to severe limitations on production and are less likely to be used for high-value crops. We do not include other provinces given the prevalence of large machinery that precludes intercropping.
Manure management	Avoided CH_4_ emissions (reported in CO_2_e) from improved management of dairy and hog manure by acidification of slurries in manure handling facilities to reduce methanogenesis. We estimate the mitigation potential of improving management of the manure from Canada’s 943,000 dairy cattle and 14.0 million swine. We focus on dairy and swine farms that could see a positive cash flow and an increase in plant- available nitrogen from implementation of the practice. We model an adoption rate by these farms of 4%/ year between 2023 and 2030.
Silvopasture (trees in agricultural lands pathway)	Increased CO_2_e sequestration from expansion of practices that integrate trees and livestock in the same area to manage simultaneously for tree crops, livestock grazing, and forage. We assume that each ruminant farm in Canada could establish an average of 20 ha of silvopasture in existing pasture lands (985,518 ha). We include carbon storage in above- and belowground biomass and soil and deductions for the albedo effect of transitioning from hay to partial deciduous cover of 111 trees/ha.
Legume crops	Avoided N_2_O and CO_2_e emissions from reduced use of nitrogen fertilizers by switching cultivation from grains to legumes. We account for avoided N_2_O emissions in the field, as well as emissions associated with manufacture of fertilizer and operation of farm machinery. We estimate the mitigation potential of transitioning 4.47 Mha of grain crops to legumes by 2030. We assess mitigation against a BAU based on recent rates of increases in legume extent.
Reduced tillage	Increased sequestration of CO_2_ in soil carbon from expanded use of no-till or reduced tillage practices in croplands. We model the expansion of no-till on 1.4 Mha and reduced tillage on 2.2 Mha of crop areas relative to a BAU scenario based on the 2016 tillage levels.
Riparian tree planting (trees in agricultural lands pathway)	Increased CO_2_e sequestration from planting trees in 30-m riparian buffers around all water bodies in agricultural zones where forests are the natural land cover (200,319 ha). We include carbon storage in above- and belowground biomass and soil and deductions for the albedo effect of transitioning from hay to full deciduous cover. To avoid double counting, we remove any overlap with the area of restoration of forest cover opportunity.
Legumes in pastures	Avoided N_2_O emissions from reduced application of nitrogen fertilizer as a result of increasing the abundance of legumes in grazed pastures. We estimate the mitigation associated with an 80% reduction in the 25.8 Gg of fertilizer applied to grazed tame pastures in British Columbia and eastern Canada. We account for avoided N_2_O emissions in the field, as well as emissions associated with manufacture of fertilizers.
Avoided conversion of shelterbelts (trees in agricultural lands pathway)	Avoided emissions of CO_2_e from aboveground biomass and of forgone carbon sequestration through avoided loss of shelterbelts across Canada’s three Prairie provinces (586 km/year). We extrapolate the observed carbon losses from shelterbelt conversion by soil zone (i.e., black, brown, dark brown, gray, and dark gray soils) and vegetation type (conifer, deciduous, and shrub) in Saskatchewan to Alberta and Manitoba based on the agricultural extent per soil zone.
Avoided wetland conversion	Avoided CO_2_e emissions of above- and belowground biomass and soil carbon due to the prevention of drainage, dredging, eutrophication, or other anthropogenic activities. We account for differences in CH_4_ and N_2_O (where relevant; see the Supplementary Materials) between converted and unconverted wetlands. We assess opportunities in peatlands (11,069 ha/year), freshwater mineral wetlands (29,335 ha/ year), and seagrass beds (1676 ha/year) as three separate pathways.
Wetland restoration	Increased sequestration of CO_2_e from restoring wetlands, through activities such as restoration of hydrological function (rewetting) or topography, moss layer transfer, fertilization, nutrient management, vegetation management, or disturbance management. We account for differences in CH_4_ and N_2_O (where relevant; see the Supplementary Materials) between restored and unrestored wetlands. We assess restoration potential in peatlands (3400 ha/year), freshwater mineral wetlands (25,000 ha/year), seagrass beds (8063 ha/year), and salt marshes (4413 ha/year) as four separate pathways.
Avoided grassland conversion	Avoided emissions of CO_2_ by preventing the conversion of 2.5 Mha of native and tame grassland and shrubland to cropland. We quantify avoided emissions from soil and roots in grassland to 30 cm of soil depth, as well as aboveground biomass in shrublands, based on the historical rates and patterns of conversion.
Riparian grassland restoration	Increased CO_2_ sequestration in soils to 30 cm depth gained by restoring cropland to grassland or shrubland in areas with severe limitations on agricultural production. We model the establishment of 30-m riparian grassland buffers around all water bodies, including prairie pothole wetlands, in all agricultural lands within areas that would not naturally support forests (265,500 ha).
Improved forest management	Additional CO_2_e storage in forests or harvested wood products relative to a BAU scenario. We model the mitigation associated with set-asides of old growth forests, enhanced forest regeneration in postharvest stands, and utilization of harvest residues (logging slash) that would have otherwise been burned for bioenergy, as well as increased use of saw logs for long-lived wood products. We assess the net mitigation of these activities independently (see the Supplementary Materials) and combined. Net mitigation includes changes in carbon storage in all forest ecosystem pools and wood products, the albedo effect of old growth set-asides and temporary land cover transitions, and substitution benefits of wood for energy and building materials.
Avoided forest conversion	Avoided CO_2_e emissions from preventing the anthropogenic conversion of forest to nonforest land use across forests in Canada. We include conversion to agriculture, oil/gas, mining, industry, forestry roads, transportation networks, municipal, and recreation development. We estimate the net mitigation benefit of avoiding the future conversion of 20,143 ha/year until 2030 against a BAU scenario, accounting for changes in albedo, as well as emissions from all forest ecosystem pools due to conversion and forgone sequestration.
Restoration of forest cover	Additional CO_2_e sequestration from restoration of forest cover with locally adapted native tree species in areas where forests historically occurred but do not currently exist because of past conversion to another land use and where regeneration of forest is not an obligation under existing forest management regimes, e.g., after forest harvest. This practice is variously termed “afforestation” in Canada’s National Inventory Report (*19*) and “reforestation” by the Intergovernmental Panel on Climate Change. Our analysis excludes tree establishment in grass-dominated biomes because these plantings are often not successful, can reduce biodiversity, and can adversely affect soil carbon. We also exclude urban areas and areas alongside major roads. To protect food security, we do not include crop and pasture lands except those with severe limitations on agricultural production. To eliminate double counting with other pathways, we do not include recently harvested forest lands, peatland areas, or areas burned by wildfire. Plantable areas are only included if they lie within 1 km of existing roads. We calculate net carbon accumulation in all forest ecosystem pools, assuming planting of 3.8 Mha between 2022 and 2030 and discounting the mitigation benefit to account for changes in albedo.
Urban canopy cover	Increased CO_2_ sequestration by increasing average tree canopy cover from 24 to 36% in Canada’s urban areas. Actions include expanding the footprint of the urban canopy while maintaining the existing canopy and compensating for tree mortality by replacing trees that die. We assess opportunities in all population centers with more than 1000 individuals and parse analyses into seven categories (industrial areas, commercial areas, institutional areas, recreational areas or parks, street trees, natural areas, and other undeveloped areas) given their different extents of canopy cover, mortality rates, management requirements, development pressures, and costs.

**Table 2:** Estimates of area involved and annual mitigation potential for NCS in Canada’s jurisdictions. For the pathways below, we provide provincial and territorial estimates of the mitigation potential and area modeled where the data are available (AB, Alberta; BC, British Columbia; MB, Manitoba; NB, New Brunswick; NL, Newfoundland and Labrador; NT, Northwest Territories; NS, Nova Scotia; ON, Ontario; PE, Prince Edward Island; QC, Quebec; SK, Saskatchewan; YT, Yukon Territories). Blank cells equal no estimated area or mitigation. For the remaining pathways, estimates are at the national level (see table S2).

**Pathway**	**Variable**	**AB**	**BC**	**MB**	**NB**	**NL**	**NT**	**NS**	**ON**	**PE**	**QC**	**SK**	**YT**	**Canada**
Cover crops	Annual mitigation in 2030 (Tg CO_2_e/ year)	2.32	0.08	1.39	0.04			0.02	1.46	0.07	0.57	3.83		9.78
	Area of opportunity (ha)	5,609,000	121,000	2,901,000	35,000	2000		17,000	1,525,000	68,000	628,000	9,578,000		20,480,000
Crop residue – biochar	Annual mitigation in 2030 (Tg CO_2_e/ year)	1.52	0.01	0.85					1.76		0.61	2.16		6.90
Crop residue – bioethanol	Annual mitigation in 2030 (Tg CO_2_e/ year)	0.91	0.01	0.38					0.37		0.13	1.12		2.92
Nutrient management	Annual mitigation in 2030 (Tg CO_2_e/ year)	1.62	0.07	1.15	0.01	0.01		0.01	0.60	0.01	0.30	2.51		6.27
Tree intercropping	Annual mitigation in 2030 (Tg CO_2_e/ year)								2.16		1.76			3.92
	Area of opportunity (ha)								438,514		358,784			797,298
Silvopasture	Annual mitigation in 2030 (Tg CO_2_e/ year)	1.23	0.12	0.21	0.001	0.001		0.001	0.12	0.001	0.06	1.09		2.83
	Area of opportunity (ha)	428,310	40,187	73,379	363	363		363	40,617	363	21,555	380,018		985,518
Reduced tillage	Annual mitigation in 2030 (Tg CO_2_e/ year)	0.20	0.01	0.25	<0.01	<0.01		<0.01	0.13	<0.01	0.06	0.24		0.91
Riparian tree planting	Annual mitigation in 2030 (Tg CO_2_e/ year)	0.12	0.01	0.03	<0.01			0.01	0.34	0.01	0.08	0.07		0.67
	Annual mitigation in 2050 (Tg CO_2_e/ year)	0.30	0.02	0.08	0.01			0.02	0.83	0.01	0.18	0.17		1.63
	Area planted (2021–2030, ha)	37,279	2,929	10,019	894			2,277	101,686	1,767	22,704	20,815		200,319
Avoided conversion of shelterbelts	Annual mitigation in 2030 (Tg CO_2_e/ year)	0.08		0.02								0.11		0.21
	Rate of conversion (km/year)	227		56								303		586
Avoided conversion of freshwater mineral wetlands	Annual mitigation in 2030 (Tg CO2e/ year)	0.5		0.3								1.7		3.1*
	Area of opportunity (ha/ year)	4,731		2,838								16,086		29,335*
Restoration of freshwater mineral wetlands	Annual mitigation in 2030 (Tg CO2e/ year)	0.1		0.04								0.2		0.4*
	Area of opportunity (ha)	41,915		26,491								134,094		250,000*
Salt marsh restoration	Annual mitigation in 2030 (Tg CO_2_e/ year)				0.51			0.55			0.44			1.50
	Area restored (ha)				15,000			16,149			12,990			44,129
Avoided grassland conversion	Annual mitigation in 2030 (Tg CO_2_e/ year)	2.90		1.31	0.30	0.01		0.25	2.75	0.10	1.63	3.41		12.7
	Rate of conversion (ha/year)	68,500		32,400	3,240	100		3,090	37,400	1,100	21,500	83,000		250,510
Riparian grassland restoration	Annual mitigation in 2030 (Tg CO_2_e/ year)	0.29		0.04								0.34		0.68
	Area restored (ha)	114,000		19,500								132,000		265,500
Improved forest management	Annual mitigation in 2030 (Tg CO_2_e/ year)	−6.07	6.47		4.13			0.73	0.44		2.19	0.03		7.92
	Old growth conserved (2021–2030, ha)	126,664	350,117		74,038			24,623	97,873		181,490	22,111		876,916
Restoration of forest cover	Annual mitigation in 2030 (Tg CO_2_e/ year)	−1.01	0.05	−0.23	0.01	0.02	0.00	0.10	0.51	0.10	0.70	−0.22	<0.01	0.05
	Annual mitigation in 2050 (Tg CO_2_e/ year)	7.36	0.93	0.80	0.48	0.06	0.01	0.22	7.04	0.21	7.40	0.34	<0.01	24.86
	Area planted (2022–2030, ha)	1,671,163	80,153	212,104	55,997	7148	1579	24,656	685,019	22,557	834,654	207,196	1424	3,807,173†

*This national estimate includes non-Prairie provinces.

†This area of restoration of forest cover opportunity includes 3523 ha identified within the National Parks of Canada that are federally managed lands.

While prior studies have examined a subset of mitigation options in Canada’s land sector (*12*, *13*), we compile, expand upon, and develop original analyses to provide the first full assessment of NCS for Canada, including freshwater and coastal marine systems. This analysis also includes previously undeveloped spatial datasets for the restoration of forest cover where trees are the natural vegetation, riparian tree planting, grassland restoration, and freshwater mineral wetland restoration, as well as agricultural land rental values and seagrass extent (see “Data and materials availability”).

While we model ambitious implementation scenarios, we constrain our estimates with safeguards for biodiversity, human needs for food and fiber, and feasibility constraints that are specific to each pathway (see the Supplementary Materials for details). Examples of safeguards for biodiversity include avoiding conversion of natural habitat to other land uses, such as native grasslands to energy crops or forests (*14*), and reliance on native tree species for restoration of forest cover. To safeguard human needs for food, our models maintain productive crop and pasture areas. On less productive lands (Canada Land Inventory class 3 lands), we model modified agricultural practices. On agricultural lands with severe limitations on agricultural production (i.e., classes 4 to 7), we modeled some conversion to treed lands. To sustain human needs for timber and forest fiber, our models maintain at least 90% of the projected BAU timber harvest. Examples of feasibility constraints include 10% implementation per year for 10 years (2021 to 2030) for most restoration and management pathways, updates in manure management infrastructure only at the end of its lifetime, planting of additional trees in agricultural lands only in provinces where standard farm machinery can accommodate trees in production areas, adoption of cover crops only where they do not conflict with cash crop production, and restoration of forest cover only within 1 km of existing roads for ease of access.

To facilitate the design of incentives to increase NCS adoption, we also quantify the mitigation potential at three price points: Canadian dollars (CAD) 10, 50, and 100 per Mg CO_2_e. This pricing is consistent with the Pan-Canadian Framework goal to increase the price of carbon from CAD 10/Mg CO_2_e in 2018 to CAD 50/Mg CO_2_e by 2022. It is also consistent with analyses showing that carbon prices of CAD 100/Mg CO_2_e or more will likely be required by 2030 to achieve the emission reductions and removals necessary to meet climate targets (*15*–*17*).

## RESULTS

**Fig. 1:**
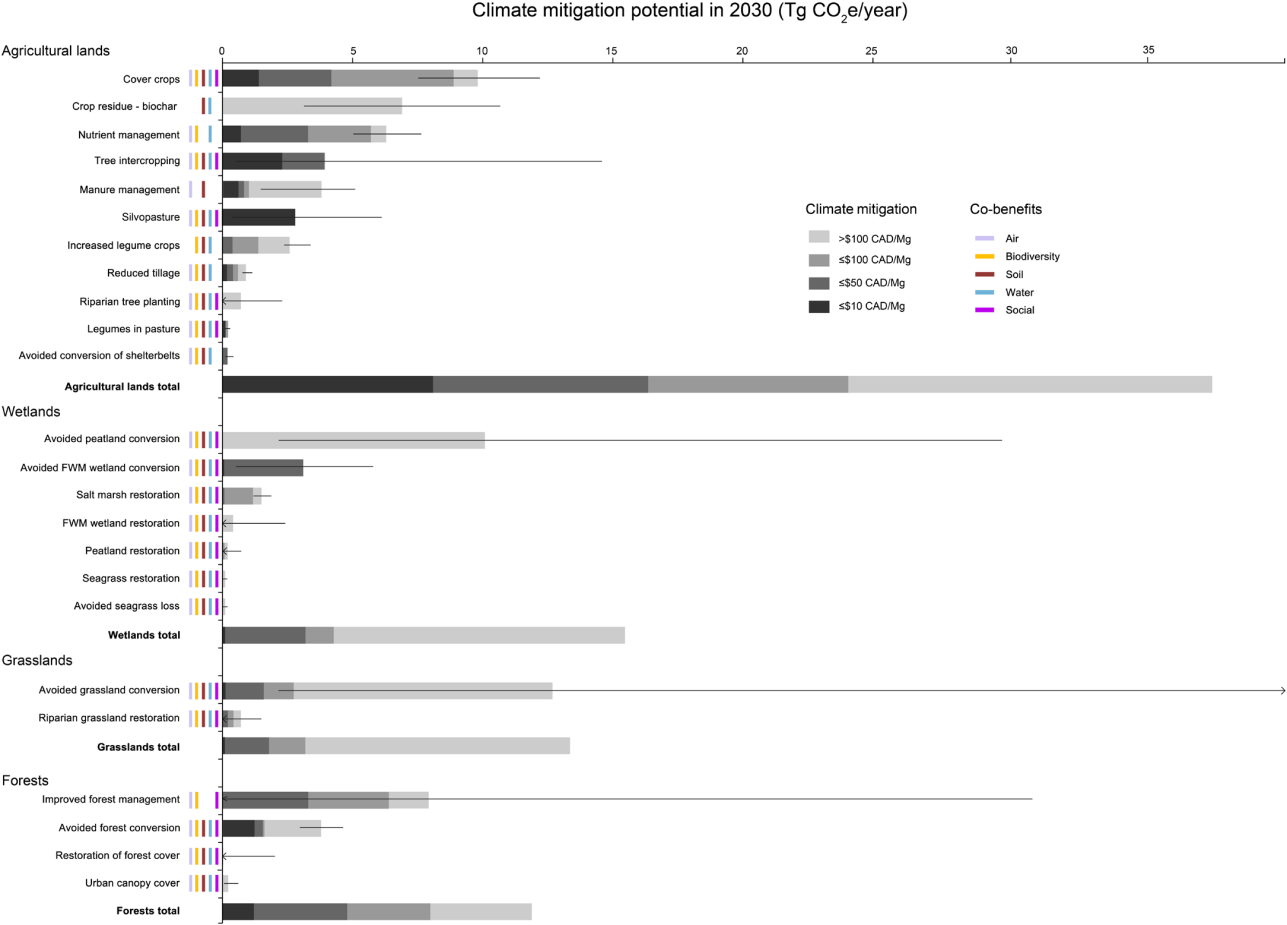
Potential annual mitigation in 2030 from 24 NCS for Canada. We indicate the mitigation potential at each price point, with lower-cost options in darker gray. Black lines indicate the 95% CI; the line with an arrow indicates where uncertainty extends beyond the graph pane (see table S2 for values). Co-benefits of each natural climate solution (table S1) are indicated by colored bars for air, biodiversity, soil, water, and social benefits. FWM wetland, freshwater mineral wetland.

In 2030, we find that the NCS can provide up to 78.2 (41.0 to 115.1) Tg CO_2_e/year [95% confidence interval (CI) shown in parentheses here and below] (Fig. 1) and cumulatively provide 394.4 (173.9 to 614.9) Tg CO_2_e of mitigation between 2021 and 2030 (Fig. 1 and table S2). Furthermore, this mitigation increases through time. Planting additional areas with trees between 2021 and 2030 (i.e., restoration of forest cover, urban canopy cover, riparian tree planting, silvopasture, and tree intercropping pathways) can provide up to 38.5 (−2.3 to 72.1) Tg CO_2_e/year of mitigation in 2050 and a cumulative total of 460.3 (221.6 to 704.0) Tg CO_2_e of mitigation between 2021 and 2050 (table S2). At CAD 10, 50, and 100/Mg CO_2_e, 12, 34, and 51%, respectively, of the total mitigation are possible in 2030 (table S2). Thus, 26.2 Tg CO_2_e/year of mitigation is available at or below Canada’s 2022 target price on carbon (CAD 50/Mg CO_2_e).

### Opportunity by pathway and sector

Avoiding conversion of grasslands, which primarily preserves soil carbon stocks, represents the largest opportunity in 2030 with 12.7 (2.2 to 41.3) Tg CO_2_e/year possible. This pathway involves preventing the conversion of 2.5 Mha of native grasslands and managed pasture to cropland between 2021 and 2030, primarily in the Prairie Pothole Region of Alberta, Saskatchewan, and Manitoba (Table 2 and fig. S3). Including the restoration of grasslands in 0.3 Mha of riparian areas brings the opportunity of grassland NCS to 13.4 (2.5 to 42.4) Tg CO_2_e/year in 2030 and 104.0 (17.4 to 348.8) Tg CO_2_e between 2021 and 2030. At CAD 50 or less per Mg CO_2_e, 13% (1.8 Tg CO_2_e/year) of the 2030 annual mitigation in grasslands is available.

Avoiding peatland disturbance from horticultural peat extraction, mine development, or road and seismic line construction represents the second largest opportunity in 2030 with up to 10.1 (2.2 to 29.7) Tg CO_2_e/year possible. This pathway is relatively expensive compared to other NCS with no mitigation available at less than CAD 100/Mg CO_2_e. However, the next largest wetland opportunity is avoided conversion of freshwater mineral wetlands, with 3.1 (0.5 to 5.7) Tg CO_2_e/year of mitigation available in 2030, all of which is available at CAD 50 or less per Mg CO_2_e. These are wetlands where at least 65% of their buffer area are cropland and thus potentially at risk of conversion. Beyond peatlands and freshwater mineral wetlands, coastal and marine opportunities (i.e., “blue carbon”) have the potential to mitigate 1.7 Tg CO_2_e/year in 2030. While avoided loss and restoration of seagrass and salt marsh habitats provide a relatively small contribution to mitigation at a national scale, their high potential to deliver NCS on a per-hectare basis and notable co-benefits warrant consideration for implementation (table S1).

Overall, wetlands can provide 15.5 (5.5 to 34.9) Tg CO_2_e/year in 2030 and cumulatively 82.6 (27.0 to 195.6) Tg CO_2_e between 2021 and 2030. Across wetland opportunities, 21% of the 2030 annual mitigation potential is available at CAD 50 or less per Mg CO_2_e (3.2 Tg CO_2_e/year).

Expanding adoption of cover crops represents the third largest NCS opportunity in 2030, which could provide up to 9.8 (7.6 to 12.1) Tg CO_2_e/year. We model adoption of cover crops on 20.5 Mha of cropland, without negatively affecting cash crop production. Cover crops can reduce net emissions by increasing soil carbon, reducing direct emissions of nitrous oxide (N_2_O), preventing nitrogen leaching and subsequent indirect N_2_O emissions, and reducing upstream emissions from fertilizer manufacturing. While most of the area of opportunity occurs in the Prairie provinces (Table 2), the highest mitigation potential per hectare is in Ontario (fig. S2).

The agriculture sector also holds other large opportunities for mitigation in 2030. Sustainably harvesting 25.7 Tg of agricultural crop residue per year and converting that residue to biochar for application to croplands can store 6.9 (3.2 to 10.6) Tg CO_2_e/year. However, we found no biochar opportunity below CAD 100/Mg CO_2_e. Improved nutrient management via adoption of the “4R” principles of fertilizer application (i.e., right source, right rate, right time, and right place) offers up to 6.3 (5.0 to 7.6) Tg CO_2_e/year of mitigation in 2030. Moreover, more than half (52%) of this opportunity is available at CAD 50 or less per Mg CO_2_e. Overall, the agricultural sector has the most mitigation potential in 2030 (37.4, 28.5 to 48.1 Tg CO_2_e/year; Fig. 1), in part, because it has more pathways than other sectors and because benefits are more immediate compared to the forest sector. Overall, 44% of the agricultural mitigation potential in 2030 is available at CAD 50 or less per Mg CO_2_e (16.4 Tg CO_2_e/year).

Forests hold the fourth largest opportunity in 2030. In particular, improved forest management offers 7.9 (−15.6 to 31.4) Tg CO_2_e/year, which in our study combines old growth conservation with enhanced forest regeneration after harvest, increased utilization of harvest residue for local bioenergy production, and a shift toward long-lived wood products (fig. S5). By 2030, we model the conservation of 0.9 Mha of old growth forests with an average age of 280 years. This activity alone (i.e., the Conservation scenario, as per the Supplementary Materials) could provide more than half (4.1 Tg CO_2_e/year) of the improved forest management opportunity in 2030. These additional old growth set-asides are distributed across Canada but occur most extensively in British Columbia, Quebec, and Alberta (Table 2). Furthermore, we find that 42% of improved forest management opportunity (3.3 Tg CO_2_e/year) is available at CAD 50 or less per Mg CO_2_e (fig. S6). Continuing implementation of this pathway between 2031 and 2050 would capture up to 27.9 (6.8 to 52.6) Tg CO_2_e/year in 2050 and cumulatively 471.4 Tg between 2021 and 2050.

By 2050, restoration of forest cover (by establishment of native tree species only where trees are the natural vegetation) represents another important opportunity in the forest sector, albeit with a low mitigation potential in 2030 of <0.1 (−2.0 to 2.0) Tg CO_2_e/year (table S2). The low 2030 mitigation potential is due to the initially slow tree growth and a delayed implementation scenario to allow time to develop planting stock (fig. S8). However, the annual mitigation potential of this pathway in 2050 [24.9 (−11.5 to 61.0) Tg CO_2_e/year] is two orders of magnitude higher than in 2030, despite no further planting in our model after 2030. Using multiple feasibility constraints, including a requirement to plant within 1 km of existing roads, we identify 3.8 Mha of potential opportunity for the expansion of forest cover (fig. S7), which would require approximately 6.3 billion trees at 1650 trees/ha (*18*).

In general, forest pathways can provide up to 11.9 (−11.7 to 35.5) Tg CO_2_e/year of mitigation opportunity in 2030 and 14.6 (−65.2 to 94.3) Tg CO_2_e cumulatively between 2021 and 2030. Furthermore, 40% of the 2030 annual mitigation from forests is available at CAD 50 or less per Mg CO_2_e (4.8 Tg CO_2_e/year), principally in improved forest management and avoided forest conversion (fig. S6).

### Protection versus improved management versus restoration

NCS related to improved management (i.e., improved forest management and most agricultural pathways; table S2) offer the highest annual mitigation potential in 2030 (44.3, 6.3 to 92.4 Tg CO_2_e/year), followed by protection (30.0, 7.9 to 81.8 Tg CO_2_e/year) and restoration (3.9, −3.6 to 11.5 Tg CO_2_e/year). The rank order of improved management, protection, and restoration remains the same when examining lower-cost opportunities, with 19.5, 6.4, and 0.3 Tg CO_2_e/year available at CAD 50 or less per Mg CO_2_e, respectively.

The relatively low mitigation value of restoration pathways in 2030, however, does not reflect the longer-term benefit of these NCS. Three restoration pathways—riparian tree planting, urban canopy cover, and restoration of forest cover with native species—show 2-fold, 8-fold, and more than 200-fold increases in annual mitigation potential by 2050 as compared to 2030, respectively. Combined, these three restoration opportunities offer 28.2 Tg CO_2_e/year in 2050 (table S2) and indicate the importance of investments in the next decade for achieving long-term climate mitigation.

### Lowest-cost opportunities

More than half of the pathways have some opportunity available at CAD 50 or less per Mg CO_2_e (table S2)—with the full opportunity available in silvopasture, tree intercropping, avoided conversion of freshwater mineral wetlands, and avoided loss of shelterbelts—and half or more of the opportunity available in cover crops, legumes in pasture, and improved nutrient management. Moreover, 12% of the annual mitigation potential (9.5 Tg CO_2_e/year) in 2030 is available at CAD 10/Mg CO_2_e. Lower-cost NCS tend to occur in locations with lower land costs (fig. S1) or have cost reductions associated with NCS implementation (e.g., reduced use of fertilizer from implementation of cover crops).

## DISCUSSION

We estimate that NCS can provide up to 78.2 Tg CO_2_e/year of mitigation potential in 2030 (Fig. 1). This potential is considerable, equivalent to the 2018 emissions from all heavy industry in Canada (*19*). Widespread implementation of NCS in ways that are additional to existing plans to reduce emissions via regulations and increased efficiencies in energy and fuel use would allow Canada to exceed its current NDC, which is critical given that mitigation efforts must increase to limit warming to 2°C (*9*). Furthermore, a third (26.2 Tg CO_2_e/year) of NCS mitigation is possible at ≤CAD 50/Mg CO_2_e, Canada’s target carbon price for 2022.

### Avoided conversion of grassland as single highest opportunity

By 2030, we find that avoiding grassland conversion and the resulting preservation of soil carbon stocks represent the single largest opportunity in Canada. The high mitigation potential stems partly from the large net area of planted and native grassland and pastures currently converted to cropland (0.25 Mha/year), according to the 2011 to 2016 data from Canada’s Census of Agriculture. During this period, cropland area in Canada expanded from 28.2 to 31.8 Mha, largely in the Prairie provinces, where it mostly replaced not only fallow lands but also grasslands used for hay and pasture (*20*). Cropland expansion from 2011 to 2016 was slower than the previous census period (2006 to 2011), suggesting that our results may be conservative.

Unlike prior NCS analyses (*7*, *8*), where forests held most of the mitigation opportunity by 2030, the relative importance of grasslands compared to forests in Canada can be partially explained by how changes in albedo due to non–tree to tree cover transitions reduced the overall mitigation benefits, especially at high latitudes and areas with seasonal snow cover. However, tree growth rates accelerate over time, and the forest sector offers the highest potential in 2050. Restoration of forest cover offers a higher opportunity than avoided grassland conversion after 2042.

### Comparison to other estimates

Depending on the pathway, our estimates of potential for the NCS in Canada are lower, similar, or higher than previous work (or in the case of coastal marine pathways, represent previously unidentified estimates). Lower estimates are partly due to our feasibility constraints and inclusion of albedo. For example, recent global studies suggest that 6.0 Mha (*7*, *21*) to 78.4 Mha (*22*) are available for restoration of forest cover in Canada based on biophysical criteria. In comparison, we estimate only 3.8 Mha of opportunity (fig. S7) after incorporating Canada-specific feasibility and more sophisticated biophysical constraints, i.e., limiting areas of opportunity to sites within 1 km of a road for ease of access and to sites with positive climate benefits by 2050 after accounting for albedo. The differences among these studies highlight the value of national-level data and analyses to assess NCS potential more accurately at a scale relevant to implementation.

Similarly, accounting for albedo lowered our estimates of mitigation potential from improved forest management relative to other studies. Smyth *et al.* (*12*) estimated that 14.5 Tg CO_2_e/year in 2021 to 2030 was possible by increasing the utilization of harvest residue for bioenergy and shifting harvests toward long-lived wood products. While there are multiple differences in modeling assumptions between our study and (*12*) (e.g., different substitution benefits and temporal window of analysis), the incorporation of albedo effects reduced our estimates of mitigation potential from 11.2 to 7.9 Tg CO_2_e/year in 2030. Conservation of old forest in this management pathway retains more carbon relative to the BAU scenario (fig. S5), even though we incorporate a lowered albedo that partly offsets the carbon storage benefit.

Our relatively higher estimates for some pathways are likely due to the incorporation of additional actions within a pathway. For example, a previous estimate of avoiding peatland conversion found 0.2 Tg CO_2_e/year of mitigation potential (*7*), whereas we estimate up to 10.1 Tg CO_2_e/year in 2030 after accounting for disturbance and conversion.

Actual mitigation potential may be even higher than what we estimated, given that there are additional actions within NCS pathways that we excluded because of limited data or high uncertainty. For example, particular types of forest management, such as fuel reduction and prescribed burning, could reduce carbon losses due to wildfire or insect disturbance, but we do not quantify this potential given the uncertainty around the extent that humans can, in practice, reduce these disturbances in Canada. In addition, many other actions with mitigation potential are possible but not considered here, such as variable retention harvesting or silvicultural systems aimed at enhancing stand structural complexity that can increase carbon stocking in working forests (*23*).

Furthermore, we focus on supply-side actions (i.e., increasing carbon storage or reducing GHG fluxes from the land sector), with analyses of only a few demand-side actions (e.g., using crop and forestry residue for bioenergy; see the Supplementary Materials). Other important demand-side strategies, such as shifting cultivation patterns toward plant-based diets, could free up more area for NCS than considered here (*24*). Additional work is needed to evaluate mitigation opportunities across both the supply side and the demand side, including their feasibility and impacts on climate, economic, and environmental goals (*25*, *26*).

### Costs of implementation

We estimate that 12% of the total 78.2 Tg CO_2_e/year is possible at CAD 10/Mg CO_2_e or less, 34% at CAD 50/Mg CO_2_e or less, and 51% at CAD 100/Mg CO_2_e. However, costs alone are insufficient for prioritizing investments in NCS. Although we estimate no mitigation potential below CAD 100/Mg CO_2_e, the soil organic carbon from peatlands cannot be recovered on meaningful time scales once lost, which argues for avoiding peatland impacts to protect irrecoverable carbon (*27*). Furthermore, some NCS offer high co-benefits that could drive implementation, even if costs are high and mitigation potential is low. For example, we identify relatively limited opportunity to capture carbon with trees in urban canopies, but additional canopy cover in cities can provide important environmental, biodiversity, and human health benefits (*28*, *29*) and deliver mitigation through reduced electricity use that is additional to what we quantify in this study (*30*). Similarly, seagrass restoration not only offers <0.1 Tg CO_2_e/year of mitigation potential in 2030 but can also support valuable commercial, recreational, and Indigenous fisheries (*31*).

The actual costs of implementation of NCS may be lower than estimated here. The land costs that we incorporate in our models assume that land will be permanently used for NCS and that financial returns from alternative land uses will be forgone indefinitely. This assumption ignores the possibility that some lands may revert to other uses if lower-cost mitigation options become available at scale (*32*).

Furthermore, NCS may be less costly on net than modeled here because they deliver ecosystem services beyond climate mitigation (e.g., clean water and flood mitigation) (table S1). These services may become increasingly valuable as climate warming progresses (*33*), and adaptation is increasingly necessary to avoid costly climate impacts. For example, building soil carbon increases the drought resilience of croplands (*34*, *35*), and protecting coastal wetlands can provide protection against storms (*36*). To the extent that funds are available for these ecosystem services, they could reduce implementation costs for landowners or land managers (*37*).

Conversely, implementation of NCS may be costlier than estimated here. For example, we did not estimate how putting large amounts of land into NCS could drive up land values (*38*). In addition, our models assume that lands at risk of conversion can be accurately targeted, but in practice, this may be difficult and could incur additional costs. As a result, implementation of avoided conversion pathways could require protecting larger areas than assumed here to fully capture all potential areas of conversion. Similarly, our models assumed that conversion could be avoided within the first year. If, in practice, more time and resources are required, then the corresponding mitigation will decrease and costs could increase. Other socioeconomic constraints, such as the willingness of individual landowners and managers to change land uses or practices, must be considered when assessing the resources needed to implement NCS at scale.

Depending on the pathway, different financial mechanisms will be needed to support implementation. For instance, relatively low-cost pathways with high certainty, such as nutrient management or reduced tillage, are more amenable to implementation using carbon offset markets. Conversely, pathways with relatively high costs or pathways with high potential but high uncertainty may be best achieved by targeted investments in proof-of-concept projects to achieve mitigation while determining ways to reduce costs or uncertainty. Alternatively, investments in NCS related to ecosystem protection may be suitable for the creation of protected areas, especially where the area of opportunity holds important or irreplaceable cultural and ecological values.

### Best practices for implementation

Beyond costs and magnitude of mitigation potential, there are other critical factors to consider when implementing NCS. Principal among these are issues related to land tenure and rights, particularly those of Indigenous peoples whose territories encompass opportunities for NCS. Inclusion of Indigenous knowledge and respect for Indigenous rights, alongside participation and consent by Indigenous peoples in the design of NCS, are necessary for successful implementation (*39*). Indigenous-led NCS provide an opportunity for reconciliation and reliance on time-proven and effective approaches for land stewardship and biodiversity conservation (*40*).

NCS can also complement efforts to conserve and restore critical habitat for biodiversity, including at-risk species. For example, the endangered maritime ringlet butterfly (*Coenonympha nipisiquit*) lives in only nine salt marshes in the world, all in the Gulf of St. Lawrence of eastern Canada (*41*). Thus, restoration of Canadian salt marshes can help support global biodiversity. However, it is possible to capture carbon in natural systems while having negative impacts on biodiversity (*42*), for example, by establishing tree cover in native grasslands (*14*). NCS, as defined in this study, preclude negative impacts to biodiversity, and implementation of individual projects must ensure that biodiversity safeguards are in place.

Last, while we do not model displacement of emissions from one jurisdiction to another (“leakage”), successful expansion of NCS will hinge on leakage being minimized through the appropriate design of sector- and landscape-level land-use planning and policies (*43*). For instance, cessation of grassland or forest conversion into agriculture will require a compensatory response in terms of societal dietary shifts or increased food production from existing agricultural lands. Recent evidence suggests that such a response is possible through investments in yield increases, closing yield gaps, diet shifts, or better spatial matching of crops to optimal growing condition (*24*, *44*). Consequently, policies aimed at mitigation through NCS must either create these conditions or thoroughly account for leakage.

### Alignment of NCS with national accounting systems

Another key consideration for implementation relates to differences between emissions and removals tracked under the United Nations Framework Convention on Climate Change (UNFCCC) and the full potential to use NCS to reduce GHG in the atmosphere. For NCS to count toward climate mitigation goals, they must be appropriately incorporated into national reporting systems.

National climate goals typically aim to reduce net emissions relative to a historical baseline. Those net emission reductions can come from avoided actions (e.g., reduced rates of forest conversion) or increased sequestration. However, country-level submissions to the UNFCCC may not include all emissions and removals from the land sector. For example, the Canadian National Inventory Report (NIR) (*19*) only quantifies emissions associated with horticultural peat extraction, which we estimate is a small fraction of the total anthropogenic peatland disturbance. Thus, much of the high mitigation potential we estimate from avoided disturbance of peatlands (i.e., from seismic lines, roads, and mine construction, in addition to horticultural extraction) would only count under national accounting rules if these peatland disturbances and the associated emissions were first reflected in the NIR. Furthermore, other wetland types beyond peatlands such as the seagrass, salt marsh, and freshwater mineral wetlands assessed here are not yet included in the NIR. This issue is broadly relevant beyond Canada. The use of NCS to tackle climate change in ways that count toward national-level climate goals requires alignment between NCS and national GHG inventory and reporting frameworks.

Areas of misalignment also highlight critical avenues of future research regarding NCS in Canada, both to improve estimates of mitigation potential and track progress toward implementation goals. Examples of pathways where such research would be useful include NCS with high mitigation potential and limited coverage in the NIR, such as rates of conversion of freshwater mineral wetlands or farmer adoption of 4R fertilizer management and cover crops.

### Uncertainties and learning by doing

Although we quantify uncertainty around the magnitude of each natural climate solution (Fig. 1 and table S2), additional uncertainty remains. We do not account for potential changes in supply or demand for agricultural and forestry products over time that would change the mitigation potential we estimate here. For example, increasing demand for wood-based pulp for packaging (currently met through reduced demand for newsprint) could counter the shift toward longer-lived wood products that we modeled. Pathway-specific analyses should seek to address these feedbacks.

We also do not model the consequences of future climate changes on Canada’s ecosystems that will influence the context and possible impact for implementation of NCS. Climate change is expected to negatively influence ecosystems by triggering, for example, drought-induced tree mortality, reduced tree growth, increased fire activity, more extreme outbreaks of bark beetles, thawing of permafrost and subsequent forest loss, sea level rise, and soil loss through increased respiration (*44*). While comprehensive analysis of climate feedbacks on NCS is beyond the scope of this paper, we focused our analyses on the near term (2021 to 2030), which we expect to largely reflect recent historical conditions.

Moreover, not all consequences of climate change present challenges for the implementation of NCS. For example, productivity in Canada’s rangelands is estimated to increase 21% by 2050 relative to the 1951-to-2006 baseline (*45*), and there is also a high likelihood that warming, increased precipitation, and higher CO_2_ will lead to higher productivity in forests where soil nitrogen and other factors are not limiting (*46*). That said, ecosystem vulnerability to climate change must be accounted for when making decisions about where and how to implement NCS. For instance, restoration of forest cover should be avoided or carefully planned (e.g., with appropriate choice of tree species) where climate change is increasing the risk of wildland fire or drought (*47*).

Targeted implementation of NCS can limit the negative impacts of climate change while additional technological solutions are in development. While NCS may marginally increase the carbon at risk from disturbance (*47*), there are large carbon stocks in Canada’s ecosystems already at risk because of unmitigated climate change (*48*). Rapid and widespread implementation of all viable climate solutions, including NCS, is needed to reduce the risk of massive carbon loss from the terrestrial biosphere (*44*). Moreover, the risk of carbon loss in any given location is quite small, with, for example, only 0.3% of Canada’s forests disturbed by wildfire or harvesting on average each year (*49*). Furthermore, NCS can increase resilience to climate impacts, e.g., rewetting wetlands can reduce the risk of peat fires (*50*).

Ultimately, coupling implementation with experimentation and monitoring would facilitate an adaptive learning approach to help resolve uncertainty around NCS and foster innovation in land management related to climate mitigation. Future analyses should also examine risks from the impacts of climate change on future emissions and opportunities to reduce those risks (e.g., from wildfire) (*47*).

In conclusion, we document high potential for mitigation of climate change from NCS in Canada. Realization of this potential in ways that complement, not substitute, decarbonization of the energy sector can help Canada exceed its NDC and help elevate global ambition around net emission reductions (*51*). Opportunities for NCS are distributed across the country (Table 2) and increase beyond 2030 (table S2). Achieving this potential depends on support from all levels of government, Indigenous peoples, landowners, and communities, as well as substantial financial investment starting as soon as possible. This study underlines how Canada has the potential to be a global leader in the adoption of NCS, by building on notable recent investments in nature-based solutions for climate and biodiversity conservation.

## MATERIALS AND METHODS

We chose 2030 as a critical target date for the implementation of NCS because (i) it is policy-relevant to Canada’s NDC, and (ii) it is distant enough to envision scaling up of mitigation action by that year but (iii) soon enough to contribute meaningfully to the urgent need for immediate mitigation of climate change. Furthermore, modeling ecosystem dynamics beyond 2030 increases uncertainty given the potential for increasing risks of natural disturbance and other climate feedbacks on mitigation potential (*47*). Typically, for restoration or management pathways, we model 10% implementation of the total opportunity extent each year between 2021 and 2030, with full implementation by 2030. However, for some pathways (see the Supplementary Materials), we delay the implementation if a 2021 start date was considered infeasible by pathway experts. For example, we assume a 2-year delay in tree planting in the restoration of forest cover and urban canopy cover pathways to allow tree nurseries time to generate sufficient planting stock. We modeled a longer implementation for improved forest management to demonstrate how initially negative cumulative mitigation by 2030 (table S2) can be overcome with continued implementation.

We examine the impact of NCS on relevant GHGs, namely CO_2_, CH_4_, and N_2_O, but provide estimates in CO_2_e to facilitate comparisons across opportunities. We also incorporate the effects of changes in albedo (reflectivity of incoming solar radiation by the land surface) for most land cover transitions that involve trees, since albedo can diminish the climate benefit of carbon storage, in particular in trees (*52*). We do not incorporate albedo into mitigation estimates for the urban canopy cover pathway because these areas have a complex energy balance that is significantly influenced by urban trees and canopy, with the ameliorating effects of urban trees on urban microclimates exceeding their albedo effect (*53*, *54*).

We consider the influence of albedo on mitigation associated with land-use change. On the basis of a spatially explicit, 0.05° resolution map of global albedo (*55*), we quantify changes in albedo and associated radiative forcing (RF) across Canada using methods of (*56*, *57*). By combining albedo data with data for monthly snow cover (*58*) and solar radiation (*59*, *60*), we obtain an estimate of prior land-use condition under blue-sky conditions and mature condition after land-use transition using the International Geosphere-Biosphere Programme system of land cover classification (*61*). The relevant land cover classes for this study include evergreen needleleaf forests, evergreen broadleaf forests, deciduous needleleaf forests, deciduous broadleaf forests, open shrubland, grasslands, cropland, barren ground, and built-up. We translate changes in blue-sky surface albedo to top-of-atmosphere (TOA) RF with four different radiative kernels (*62*–*64*).

To convert albedo-induced RF to CO_2_e, we compute the CO_2_e flux per square meter that would have an RF equal to the mean annual TOA RF resulting from albedo change. We achieve this computation by inverting the CO_2_ RF equation to solve for the additional atmospheric loading (emission or removal) of CO_2_ that corresponds to the albedo-induced TOA RF for each land cover conversion. We adopt the global annual mean RF caused by carbon emissions per square meter of global surface area from (*65*), describing a perturbation to Earth’s TOA radiation budget imposed by a change in global atmospheric CO_2_ concentration. We then divide the corresponding CO_2_ emissions (or uptake) by grid cell area to obtain the equivalent carbon mass flux per square meter. Each year’s albedo-induced equivalent CO_2_ flux includes only the year-over-year change in albedo-induced RF. Positive fluxes represent land uptake of CO_2_ when the difference in RF in 1 year to the next is >0, emissions to the atmosphere when this difference is <0, and no flux when there is no change in RF from 1 year to the next. This method is analogous to the time-dependent emissions equivalent approach (*66*). Last, we use the median CO_2_e for all pixels located within each ecoregion and average across all four radiative kernels (at the 50th percentile of observed snow cover) to derive a mean and SD estimate of the albedo effect for all relevant land-use transitions.

We do not consider oceanic or land uptake of CO_2_ emitted by the land cover change nor the possible ocean or land release that would be induced by CO_2_ removals from land cover change. We do not consider impacts of land cover change on evapotranspiration or cloud cover. Although they can be important in some regions of the globe (*67*), these effects tend to be modest at the high latitudes studied here (*68*). We assume that the climate impact of CO_2_ emissions and removals are time independent and persist in perpetuity. In reality, the land and oceans modulate CO_2_ emissions and removals, with net absorption of a portion of emitted CO_2_ (the so-called airborne fraction is what remains) or with net release of CO_2_ partially offsetting a fraction of CO_2_ removals from the atmosphere. We assume equal efficacy between the TOA RF from CO_2_ and the TOA RF from surface albedo changes, while recognizing that effects on surface temperature can have distinct spatial patterns across the globe (*69*).

For each pathway, as well as aggregations of pathways (e.g., all forest pathways), we estimate uncertainty around the total annual mitigation potential in 2030. To do so, we incorporate uncertainty around the extent of opportunity (i.e., hectares, heads of cattle, and tons of fertilizer) and flux (i.e., additional accumulation or reduced emissions per unit of extent) or around overall mitigation. Unless otherwise noted, we calculate uncertainty with Monte Carlo simulations with 100,000 iterations. We incorporate uncertainty around individual parameters in as disaggregated a form as possible. For example, for albedo, we had separate estimates of uncertainty per ecoregion, whereas for urban canopy cover, we had a single estimate of uncertainty around potential rates of carbon accumulation. Depending on the variable, we fit the data with a normal distribution, a log-normal distribution if the values could not go below zero (e.g., biomass accumulation or area of opportunity), or a triangular distribution. Throughout the Supplementary Materials, we describe any variations on the above approach. For mitigation estimates, as well as individual model parameters, we note the lower and upper bound of the 95% CI in parentheses since uncertainty was often asymmetric around the mean. We also note the units for any uncertainty estimates that do not represent 95% CI.

For each pathway, we also construct marginal abatement cost (MAC) curves from intervention costs and abatement estimates. A MAC curve represents the monetary cost of achieving successive units (e.g., Mg CO_2_e) of sequestered or avoided GHG emissions and shows the total quantity of net emission reductions that can be achieved at different price points. We consider implementation costs, opportunity costs, and, where available, avoided on-site costs from implementation of NCS. We assume that landowners or managers would be willing to implement interventions if they are compensated for their full net costs associated with implementation. For most pathways, our cost estimates do not capture the full transaction costs as this information is rarely available and these costs depend on the type and design of implementation mechanisms (*70*). Transaction costs are typically not included in cost estimates of large-scale GHG mitigation, so their partial omission in our study does not necessarily bias comparisons with cost estimates from other mitigation studies. We highlight primarily the mitigation possible at CAD 50 or less per Mg CO_2_e given the Pan-Canadian Framework goal to reach this price on carbon by 2022.

For all pathways, we use a 3% discount rate for costs and 1% discount rate for mitigation. The 3% discount rate for costs represents the Treasury Board of Canada Secretariat’s suggested social rate for cost-benefit analyses (*71*). We adjust all dollars using appropriate price indexes to allow reporting in 2018 CAD. We discount carbon, since there is a greater social value from mitigation now rather than later (*72*) and the discount rate for mitigation (*72*) approximates how the marginal damage of one additional unit of CO_2_e emitted to the atmosphere increases over time (*73*, *74*). The 1% discount rate stems from a 2% annual growth rate of the social cost of carbon (*75*), subtracted from the 3% social discount rate (*76*, *77*) for costs.

For several pathways, we estimate the opportunity costs of forgone revenue from the implementation of NCS based on agricultural land rental values (fig. S1). We derive these values using data from Statistics Canada’s 2011 Census of Agriculture for agricultural rental expenses (*20*) and land tenure (*78*) at the scale of the Census Consolidated Subdivision (CCS), the most detailed spatial scale available. We use the 2011 data because the 2016 data have coarser cost estimates and spatial resolutions. We exclude Canada’s northern territories from the calculation of agricultural land values as data were sparse and collected with a different methodology. For the remaining CCS with missing data, we estimate an average value based on the five nearest spatial neighbors to generate a complete map of agricultural rent values. We define option value as the value of maintaining the possibility to switch land use between different uses. As reliable data are not available to represent marginal lands with relatively low rental payments, we assume that the annual average rental payments in the above land value calculations capture the option value. Our estimates of rental payments are for all levels of farmland rather than marginal farmland only and may therefore overestimate the forgone land value for the implementation of NCS in marginal farmlands.

## SUPPLEMENTARY MATERIALS

Supplementary material for this article is available at http://advances.sciencemag.org/cgi/content/full/7/23/eabd6034/DC1
